# The Gut Microbiota Modulates Glycaemic Control and Serum Metabolite Profiles in Non-Obese Diabetic Mice

**DOI:** 10.1371/journal.pone.0110359

**Published:** 2014-11-12

**Authors:** Thomas U. Greiner, Tuulia Hyötyläinen, Mikael Knip, Fredrik Bäckhed, Matej Orešič

**Affiliations:** 1 The Wallenberg Laboratory and Sahlgrenska Center for Cardiovascular and Metabolic Research, Department of Molecular and Clinical Medicine, Institute of Medicine, University of Gothenburg, Gothenburg, Sweden; 2 VTT Technical Research Centre of Finland, Espoo, Finland; 3 Steno Diabetes Center, Gentofte, Denmark; 4 Children's Hospital, University of Helsinki and Helsinki University Central Hospital, Helsinki, Finland; 5 Diabetes and Obesity Research Program, University of Helsinki, Helsinki, Finland; 6 Folkhälsan Research Center, Helsinki, Finland; 7 Department of Pediatrics, Tampere University Hospital, Tampere, Finland; 8 Novo Nordisk Foundation Center for Basic Metabolic Research, Section for Metabolic Receptology and Enteroendocrinology, Faculty of Health Sciences, University of Copenhagen, Copenhagen, Denmark; Children's Hospital Boston/Harvard Medical School, United States of America

## Abstract

Islet autoimmunity in children who later progress to type 1 diabetes is preceded by dysregulated serum metabolite profiles, but the origin of these metabolic changes is unknown. The gut microbiota affects host metabolism and changes in its composition contribute to several immune-mediated diseases; however, it is not known whether the gut microbiota is involved in the early metabolic disturbances in progression to type 1 diabetes. We rederived non-obese diabetic (NOD) mice as germ free to explore the potential role of the gut microbiota in the development of diabetic autoimmunity and to directly investigate whether the metabolic profiles associated with the development of type 1 diabetes can be modulated by the gut microbiota. The absence of a gut microbiota in NOD mice did not affect the overall diabetes incidence but resulted in increased insulitis and levels of interferon gamma and interleukin 12; these changes were counterbalanced by improved peripheral glucose metabolism. Furthermore, we observed a markedly increased variation in blood glucose levels in the absence of a microbiota in NOD mice that did not progress to diabetes. Additionally, germ-free NOD mice had a metabolite profile similar to that of pre-diabetic children. Our data suggest that germ-free NOD mice have reduced glycaemic control and dysregulated immunologic and metabolic responses.

## Introduction

Type 1 diabetes is an autoimmune disorder characterized by the specific destruction of insulin-producing beta cells in the pancreas [1]. The incidence of type 1 diabetes among children and adolescents has increased markedly in western countries in recent decades [2]. In contrast to other autoimmune diseases with a clear female bias, type 1 diabetes has no clear gender separation in children and has a male bias in young adults [3]. Genetic risk factors affect disease incidence; however, less than 10% of the individuals carrying human leukocyte antigen (HLA) risk alleles develop diabetes and the concordance between monozygotic twins is less than 40%, suggesting that environmental factors are important for disease development [4], [5]. Infections as well as dietary factors have been implicated as triggers of autoimmunity and disease progression [6] and, according to the hygiene hypothesis, infections early in life could prevent allergic diseases [7]. Despite promising results in rodent models, clinical trials to treat type 1 diabetes using immunotherapy such as anti-CD3 monoclonal antibodies or cytotoxic T lymphocyte antigen-immunoglobulin (CTLA4-Ig) have only shown a partial improvement of the disease [8]–[10]. Recent trials using haematopoietic stem cell transplantation as a treatment of type 1 diabetes have shown promising results but it is still unclear whether it can safely be used to treat all patients [8]. Thus, there is still a need to identify new factors that trigger type 1 diabetes that may be targeted in treatment of the disease.

The gut microbiota is a complex microbial ecosystem that is considered to be a major driving force in immunological maturation [11]. Its profound role in regulating host metabolism has become evident in recent years [12] and emerging evidence indicates that it is a possible modulator of diabetes. Several reports have demonstrated that an altered microbiota is associated with risk of developing type 2 diabetes [13], [14] and both preclinical and clinical type 1 diabetes [15], [16]. Furthermore, maternal use of phenoxymethyl or quinolone antimicrobials before pregnancy is associated with an increased risk of type 1 diabetes in the offspring [17], [18]. Studies in non-obese diabetic (NOD) mice suggest a direct involvement of the gut microbiota in the development of autoimmune diabetes [19], [20]. However, the mechanisms that link the microbiota with autoimmune disease remain to be determined.

A recent report suggested that microbially driven increases in testosterone levels can explain the relative protection against diabetes observed in male NOD mice [21]. However, because puberty does not confer protection against type 1 diabetes in men [3], it is unclear whether this microbially derived effect is present in humans and suggests that there are additional effects of the microbiota on type 1 diabetes development that are not gender dependent.

Changes in the microbiota have been shown to affect the metabolome of the host and our previous metabolomics study revealed that metabolic abnormalities precede islet autoimmunity in children who later progress to type 1 diabetes independent of HLA-associated genetic risk [22]. We also showed that lipidomic profiles of children who progress to type 1 diabetes are similar to those of female NOD mice [23]. Here we rederived NOD mice as germ free (GF) to investigate the microbial influence on diabetic autoimmunity and to directly investigate whether the metabolic profiles associated with the development of type 1 diabetes can be modulated by the gut microbiota.

## Materials and Methods

### Mice

GF NOD mice were rederived at Taconic farms and were thereafter maintained in flexible plastic film isolators under a strict 12-h light cycle (lights on at 06:00 h) at the University of Gothenburg. GF status was verified regularly by anaerobic culturing in addition to PCR for bacterial 16S rDNA. Both GF and conventionally raised (CONV-R) NOD mice were fed autoclaved chow diet (Labdiet) *ad libitum*. Diabetes incidence was determined by monitoring blood glucose levels weekly by sampling of a small volume of blood from the tail vein. Mice were considered diabetic when values exceeded 18 mM for two consecutive weeks or 25 mM for one week. Blood for metabolomics and cytokine analyses was collected from the *vena cava* at the termination of the experiment under deep isoflurane anesthesia after a 4 h fast, unless otherwise stated. This study was carried out in accordance with the recommendations for Laboratory Animals in Sweden. The protocol was approved by the Committee on the Ethics of Animal Experiments at the University of Gothenburg (Permit Number: 338–2012). All efforts were made to minimize animal suffering.

### Insulitis and beta cell area

Sections 200–300 µm apart from the pancreas of 4-, 9- and 23-week-old mice were stained with haematoxylin and eosin and scored according to the following criteria: no infiltration (0); peri-insulitis (1); ≤50% destruction of islet (2); ≥50% destruction of islet (3). We scored 100 (at 4 and 9 weeks) and 50 (at 23 weeks) islets per mouse. For beta cell area, sections 300 µm apart through the whole pancreas were stained with guinea pig anti-insulin (Dako), visualized using VECTASTAIN ABC and Vulcan Fast Red Chromogen Kit 2 (Histolab), and counterstained with haematoxylin. The area stained for insulin was compared with the total area of the tissue using Biopix software.

### Insulin autoantibodies

Serum insulin autoantibodies (IAA) were analysed using a competitive radiobinding assay as previously described [15]. The results were expressed in relative units (RU) based on standard curves run on each plate. The cut-off value for mouse IAA positivity was set at the mean+3SDS in 29 BALB-mice, i.e. 0.88 RU.

### Cytokine measurements

Serum from mice at 15 and 23 weeks was analysed with Mesoscale 9-plex cytokine assay according to the manufacturer's instructions.

### Oral glucose tolerance test (OGTT) and insulin measurements

Mice aged 17–18 weeks were fasted for 4 h and orally gavaged with 20% D-glucose (2 g/kg). Blood was drawn from the tail vein at 0, 15, 30, 60, 90 and 120 min and blood glucose levels were measured using a HemoCue glucometer. Insulin levels were measured at 0, 15 and 30 min after gavage. In mice that did not progress to diabetes by 30 weeks of age, insulin levels were measured after a 4 h fast. Insulin was measured using insulin ELISA-kit (Crystal Chem).

### Analysis of polar metabolites by GC×GC-TOFMS

An established metabolomics platform using two dimensional gas chromatography coupled to time-of-flight mass spectrometry (GC×GC-TOFMS) was used to analyse polar metabolites in serum [24]. Serum samples (30 µl) were combined with 10 µl of an internal standard, labelled palmitic acid (16:0–16,16,16d3; 500 mg/l), and 400 µl of methanol, vortexed for 2 min and incubated for 30 min at room temperature. The supernatant was separated by centrifugation at 5590 *g* for 5 min at room temperature. The sample was dried under a constant flow of nitrogen. Twenty-five µl of 2% methoxyamine hydrochloride in pyridine was added to the dried sample and incubated at 45°C for 1 h and then derivatized with 25 µl of N-methyl-N-(trimethylsilyl)-trifluoroacetamide by incubating at 45°C for 1 h. Five µl of retention index standard mixture with five alkanes (400 mg/l) was added to the metabolite mixture. Sample order for analysis was established by randomization. The samples were analysed on a Leco Pegasus 4D GC×GC-TOF mass spectrometer with Agilent technologies 6890N GC and Combi PAL autosampler. Data were processed using the Guineu software [24].

### Analysis of molecular lipids by UPLC-MS

An established platform based on Acquity Ultra Performance LC coupled to time-of-flight mass spectrometry (UPLC-MS) was used to analyse the molecular lipids in aliquots (10 µl) of serum samples [25]. The data were processed using MZmine 2 software [26] and the lipid identification was based on an internal spectral library or on *de novo* identification using tandem MS [25].

### Statistical analysis

Data were analysed by Student's t test and presented as mean ± SEM or SD. Survival curves were analysed with log-rank (Mantel-Cox) test. IAA positivity was analysed by Fisher's exact test. Univariate statistical analysis of metabolomics data used MATLAB r2012a. Clustering was performed and visualized using the MeV software [27].

## Results

### Colonization status affects insulitis and the presence of IAA in NOD mice

**Figure 1:**
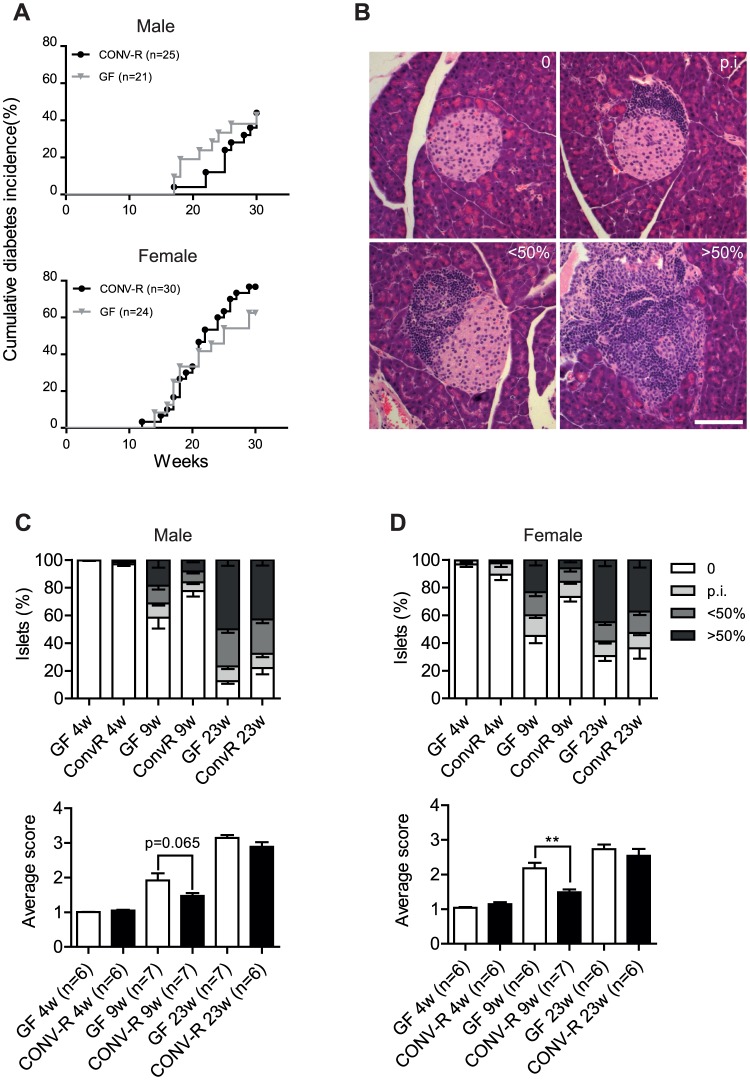
Diabetes incidence and insulitis in NOD mice. (A) Cumulative diabetes incidence in male and female GF and CONV-R mice. (B) Representative images of islets in four insulitis categories: 0 (no infiltration); p.i. (peri-insulitis); <50% destruction; >50% destruction. Scale bar represents 100 µm. (C, D) Distribution of islet scores and average insulitis score in GF and CONVR male (C) and female (D) mice at 4, 9 and 23 weeks (n = 6–7). Data are presented as mean ± s.e.m. **p<0.01 determined by Student's *t*-test.

To investigate the potential role of the gut microbiota in the development of type 1 diabetes, we rederived NOD mice as GF. Female NOD mice developed diabetes at a higher rate and at an earlier time point than male NOD mice (Fig. 1A), in agreement with a previous report [28]. Disease onset was earlier in GF compared with CONV-R male NOD mice but no difference was observed between female GF and CONV-R NOD mice (Fig. 1A). There was no significant difference between diabetes progression of GF and CONV-R NOD mice (both male and female) by 30 weeks (Fig. 1A).

To further investigate autoimmunity in the NOD mice, we investigated the rate of islet infiltration (Fig. 1B). At 9 weeks, there was a tendency towards enhanced insulitis in male GF NOD mice and a significant increase in insulitis in female GF NOD mice compared with their CONV-R NOD counterparts (Fig. 1C, D). The difference in rate of infiltration was only transient since there was no difference in insulitis score between GF and CONV-R NOD mice that had not yet progressed to diabetes at 23 weeks (Fig. 1C, D). In addition, we did not observe any differences in beta cell area of the pancreas in female NOD mice at 23 weeks (Fig. S1).

We observed that GF NOD mice were significantly more likely to be positive for IAA compared with CONV-R NOD mice at 4 weeks (Table 1) and autoantibody titers were higher in GF compared with CONV-R NOD mice at 9 weeks (Fig. S2).

**Table 1:** Proportion of insulin autoantibody (IAA)-positive mice in relation to age.

Age			P value
	GF	CONV-R	
4 weeks	76.5% (n = 17)	38.9% (n = 18)	0.041
9 weeks	92.9% (n = 14)	61.9% (n = 21)	0.056
23 weeks	90% (n = 20)	75% (n = 16)	0.374

Analysis of serum samples of germ-free (GF) and conventionally raised (CONV-R) NOD mice. Data were analyzed by Fisher's exact test.

### Colonization status affects serum cytokine levels

**Figure 2:**
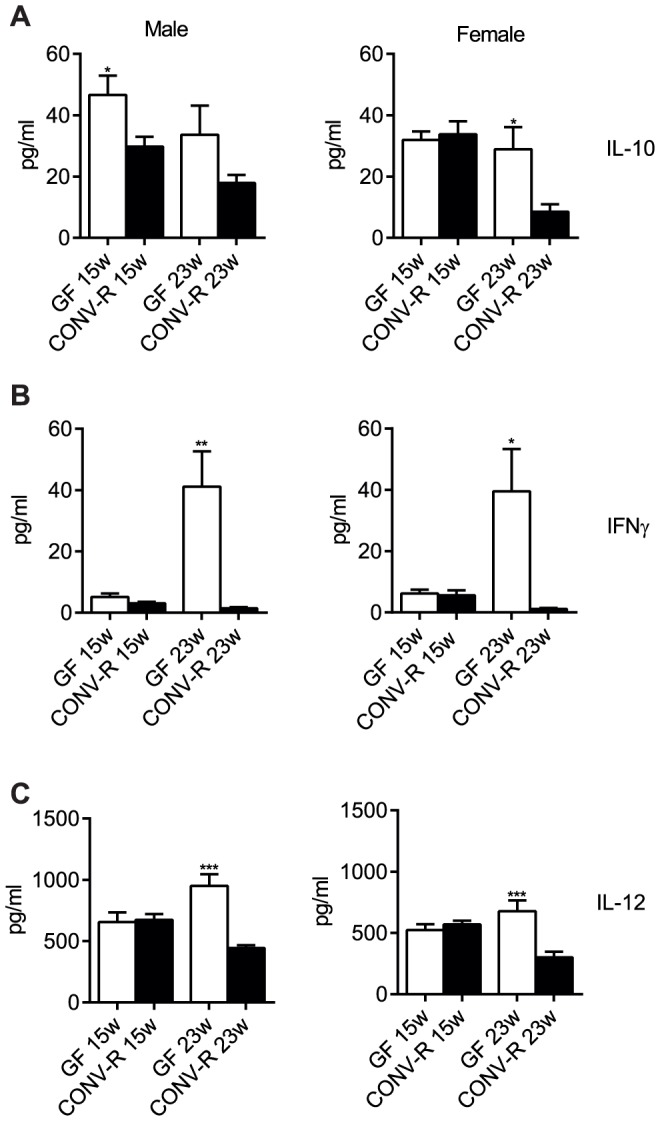
Altered serum levels of pro- and anti-inflammatory cytokines in GF NOD mice. Serum levels of IL-10 (A), interferon gamma (B) and IL-12 (C) in male and female mice at 15 and 23 weeks. Data are presented as mean ± s.e.m. *p<0.05, **p<0.01, ***p<0.001 GF vs CONV-R mice determined by Student's *t*-test. (Female: 15 weeks n = 9; 23 weeks n = 5. Male: 15 weeks n = 6; 23 weeks n = 7.).

To analyse potential changes in immunological parameters in response to the absence of microbiota, we analysed interleukin 10 (IL-10), interferon gamma (IFN-γ) and interleukin 12 (IL-12). At 15 weeks, IL-10 levels were higher in GF versus CONV-R male NOD mice, but no differences were noted for IFN-γ and IL-12 (Fig. 2A–C). However, at 23 weeks, levels of IFN-γ and IL-12 were significantly higher in male and female GF NOD mice compared with their CONV-R counterparts (Fig. 2B, C) and IL-10 levels were reduced in female CONV-R mice (Fig. 2A).

### Improved peripheral glucose metabolism in GF NOD mice

**Figure 3:**
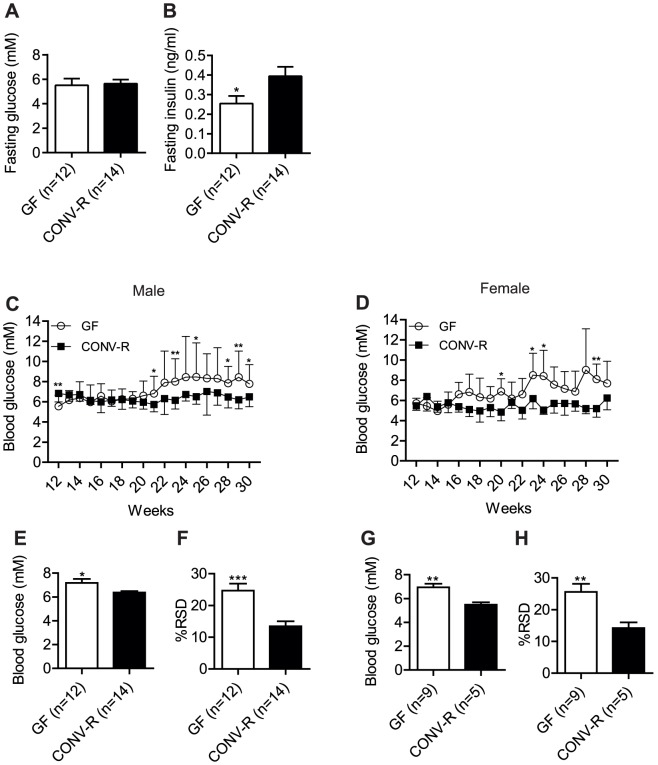
Altered blood glucose control in GF NOD mice. (A,B) Fasting blood glucose (A) and insulin levels (B) in male non-progressing mice 30 weeks of age. (C,D) Non-fasting blood glucose levels in non-diabetic male (GF n = 12, CONV-R n = 14) (C) and female (GF n = 9, CONV-R n = 5) (D) mice from 12–30 weeks. (E–H) Average blood glucose levels per mouse and percent relative standard deviation (RSD) of glucose values per mouse from 12–30 weeks in male (E,F) and female (G,H) mice. Data are presented as mean ± s.e.m. (A, B, E–H) or mean ± s.d. (C,D). *p<0.05, **p<0.01, ***p<0.001 vs CONV-R mice determined by Student's *t*-test.

We hypothesized that peripheral glucose metabolism is improved in NOD mice lacking a microbiota, which may partly explain why we did not observe an increased rate of diabetes incidence in GF NOD mice despite an increase in IAA and pro-inflammatory cytokines compared with CONV-R NOD mice. At 30 weeks, fasting glucose levels were not different between GF and CONV-R non-progressing male NOD mice, but fasting insulin levels were lower in the GF mice (Fig. 3A, B), indicating an improved peripheral glucose metabolism similar to GF wild-type mice on different genetic backgrounds [29], [30]. We also performed an OGTT in GF and CONV-R NOD mice at 17–18 weeks of age (in the total group, i.e. both non-progressors and progressors). We observed a faster glucose clearance in GF versus CONV-R female NOD mice and a tendency towards improved glucose tolerance in GF versus CONV-R male NOD mice (Fig. S3). Insulin levels after an OGTT were not different between GF and CONV-R NOD mice (Fig. S3), indicating that the improved glucose clearance in GF NOD mice was not due to enhanced insulin secretion.

### Dysregulation of blood glucose in non-progressing GF NOD mice

Although we did not see any difference in diabetes incidence between GF and CONV-R NOD mice, we observed striking differences when we analysed the non-fasting blood glucose levels in the mice that did not progress to diabetes by 30 weeks of age (Fig. 3C–H). At 12 weeks, blood glucose levels were lower in non-progressing GF NOD male mice compared with their CONV-R counterparts (Fig. 3C). However, from 20 weeks, blood glucose levels were significantly higher in non-progressing GF compared with CONV-R NOD mice (Fig. 3C, D). The mean blood glucose levels and the percent relative standard deviation (%RSD) in blood glucose levels per mouse from 12–30 weeks were higher in non-progressing GF compared with CONV-R NOD mice (Fig. 3E–H), further demonstrating the decrease in blood glucose control in both male and female GF NOD mice.

### Colonization status affects the serum metabolic profile in NOD mice

**Figure 4:**
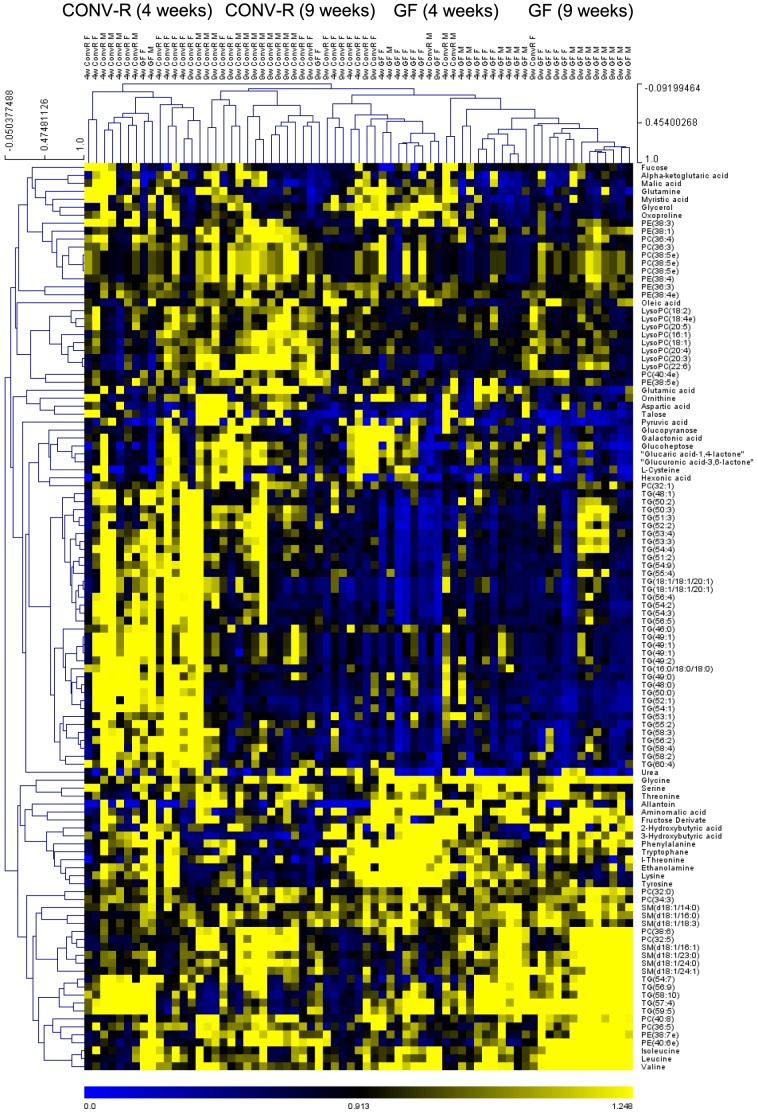
Heatmap of significantly altered identified metabolites in GF and CONV-R NOD mice in 4- and 9-week old mice. For each metabolite, the levels in each mouse are normalized to the mean level in CONV-R mice, i.e., yellow and blue indicate increased and decreased levels of the metabolites, respectively, relative to the average level in CONV-R mice. Hierarchical clustering is applied across all metabolites (performed across all n = 69 samples analyzed) and all mice. (Female n = 6–10. Male n = 7–11.).

To investigate whether the early metabolic abnormalities preceding islet autoimmunity in children who progressed to type 1 diabetes observed in a previous human study [22] might be modulated by the gut microbiota, we performed global metabolomic analysis of serum taken from GF and CONV-R NOD mice (both non-progressors and progressors to autoimmune diabetes). Two analytical platforms with broad analytical coverage were applied to all samples: (1) a global lipidomics platform based on UPLC-MS, which covers molecular lipids such as phospholipids, sphingolipids, and neutral lipids; and (2) a platform for global profiling of small polar metabolites based on comprehensive two-dimensional GC×GC-TOFMS, which covers small molecules such as amino acids, free fatty acids, keto-acids, various other organic acids, sterols, and sugars. A total of 358 molecular lipids and 195 polar metabolites were detected and included in the analysis. When observing the significantly altered metabolites in young mice, we found that the colonization status was the dominant factor that affected the clustering of metabolic profiles (Fig. 4), in agreement with recent findings by Markle et al. [21]. Within the GF and CONV-R groups, we observed subclustering by age and gender (Fig. 4).

**Figure 5:**
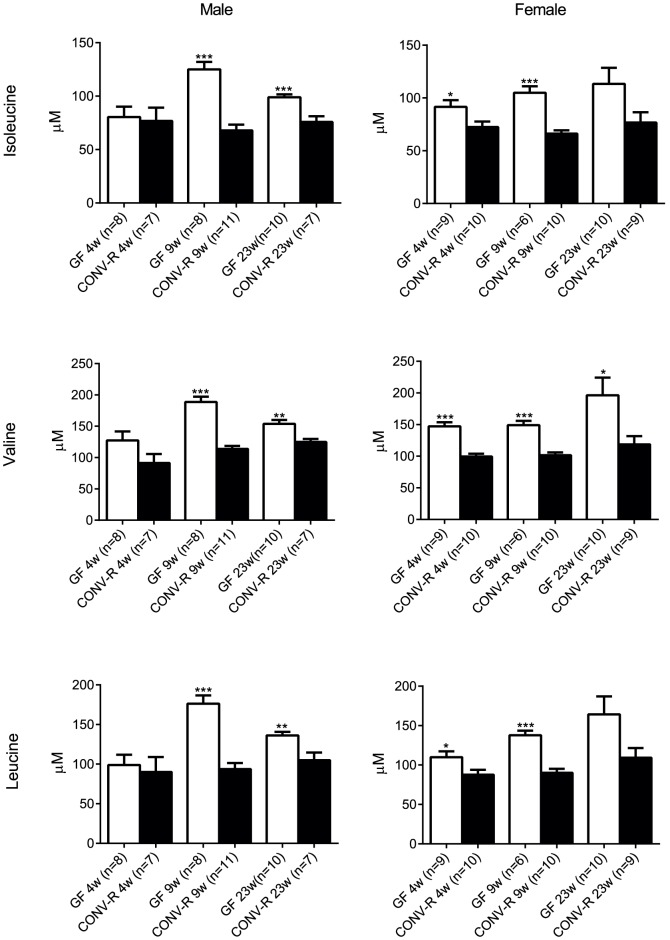
Increased levels of branched chain amino acids in GF NOD mice. Relative concentrations of branched chain amino acids mice at 4, 9 and 23 weeks. (Male n = 7–11. Female n = 9–14.) Data are presented as mean ±s.e.m. *p<0.05, **p<0.01, ***p<0.001 vs CONV-R mice determined by Student's *t*-test.

The colonization status had a similar effect on metabolic profiles of 4- and 9-week-old mice (r = 0.52, P<0.001, for the associations of CONV-R/GF fold changes in 4- and 9-week old mice for the 156 significantly different metabolites). As expected, the metabolites of similar structural class tended to co-cluster (Fig. 4), thus allowing for comparison of metabolic profiles at the class level. GF NOD mice had reduced levels of tricarboxylic acid (TCA) cycle metabolites, sugar derivatives and triglycerides compared with CONV-R mice (Fig. 4). Furthermore, branched chain amino acids (BCAAs) were elevated at 9 and 23 weeks in male GF NOD mice and at 4 and 9 weeks in female GF NOD mice (Figs. 4, 5). There was also an elevated cluster of metabolites in the profile of GF NOD mice that consisted primarily of amino acids including glycine, phenylalanine, tyrosine and 2-hydroxybutyric acid (Fig. 4).

We previously observed that differences between the cord serum metabolomes of newborn infants who progress to type 1 diabetes and their controls are similar to differences found between the 9-week-old GF and CONV-R Swiss Webster mice [31]. To determine whether these similarities were also observed in the NOD mice, we compared previously acquired human data from 15 type 1 diabetes progressors and 24 controls [22] with the metabolomics dataset from 9-week-old GF and CONV-R NOD mice. Triglycerides were excluded from the comparison due to high variability in the clinical dataset. A total of 53 lipids and 25 polar metabolites were matched across the datasets. We found that the difference in the metabolome of type 1 diabetes human progressors compared with controls significantly correlated with that of GF compared with CONV-R NOD mice (r = 0.43, P = 7.0×10^−5^, comparing mean concentrations of each of the 78 metabolites in type 1 diabetes progressors and GF mice, scaled by mean concentrations in non-progressors and CONV-R mice, respectively).

## Discussion

Here we identified a profound role of the gut microbiota in the regulation of glycaemic control as well as of immunological and metabolic profiles in a mouse model of autoimmune diabetes. The absence of gut microbiota in NOD mice did not affect overall disease incidence, but resulted in increased insulitis and levels of IAA and pro-inflammatory cytokines, and worsened glucose control in mice that did not progress to diabetes. Furthermore, we observed differences in the serum metabolomic profiles between GF and CONV-R NOD mice that resembled the difference between those of newborn infants who later progress to type 1 diabetes and non-progressors, suggesting that the metabolomic changes in infants who later progress to type 1 diabetes may be modulated by the gut microbiota.

The similarity in diabetes incidence in both male and female mice between GF and CONV-R NOD is in agreement with earlier studies [21], [32]. We observed an increase in insulitis in GF NOD mice at 9 weeks, again consistent with previous results [32], but the effect was only transient and there was no difference in beta cell area in older mice. Disease incidence is affected by housing conditions [33] and the relatively high disease incidence in our CONV-R mice could be explained by the clean housing conditions in our specific pathogen free facility. IAA positivity was more frequent at 4 weeks of age in GF versus CONV-R NOD mice, which may mirror progressive beta cell autoimmunity [34] and contribute to the enhanced beta cell destruction indicated by the increased insulitis at 9 weeks. The gut microbiota is known to affect B-cell development [35] and B-cells have been shown to be important contributors to diabetes progression in the NOD mouse model [36]. However, the difference in IAA production from B-cells did not contribute to an effect on overall diabetes incidence.

We observed that levels of IFN-γ and IL-12 were higher in GF compared with CONV-R NOD mice at 23 weeks in both male and female mice. These cytokines are known to influence diabetes progression in NOD mice [37], and IL-12-induced secretion of IFN-γ at the effector stage of diabetes development has previously been shown to promote diabetes in NOD mice [38], [39]. The increase in IL-12 and IFN-γ at 23 weeks was paralleled by an increase in autoantibody titers in GF NOD mice, consistent with an association between these cytokines and autoimmunity. In addition, we observed a decrease in IL-10 levels in female CONV-R at 23 weeks of age. IL-10 has been shown to protect against diabetes in NOD mice but only when administered from an early age [40], and thus the reduction in IL-10 levels we observed might not have an influence on autoimmunity.

Earlier studies have shown that GF wild-type mice have improved peripheral glucose metabolism compared with their CONV-R counterparts [30]. We therefore tested the hypothesis that the incidence of diabetes is similar in GF and CONV-R NOD mice because improved peripheral glucose metabolism counterbalances the increased autoimmunity in GF NOD mice. In agreement with our hypothesis, we showed that fasting insulin levels in NOD mice that had not progressed to diabetes by 30 weeks were lower in GF versus CONV-R mice despite similar fasting glucose levels. Furthermore, female GF NOD mice had improved glucose clearance at 17–18 weeks. We propose that the GF NOD mice are more resistant to developing diabetes because lower levels of insulin are required to maintain normoglycaemia in these mice. However, the blood glucose levels of the GF NOD mice that had not progressed to diabetes by 30 weeks increased after 20 weeks of age with considerably higher variability compared with CONV-R NOD mice in both male and female mice, suggesting that blood glucose control in the non-fasting state is reduced in GF NOD mice.

We observed differences in the global profiles of polar metabolites and molecular lipids between 9-week-old GF and CONV-R NOD mice that were similar to those reported earlier between GF and CONV-R Swiss Webster mice [31]. Specifically, as previously seen in GF wild-type Swiss Webster mice, GF NOD mice had reduced levels of TCA cycle metabolites, sugar derivatives and triglycerides compared with their CONV-R counterparts. However, in contrast to an increase in only one BCAA (valine) in GF versus CONV-R Swiss Webster mice [31], we observed an increase in all three BCAAs (isoleucine, valine and leucine) in GF versus CONV-R NOD mice irrespective of gender. Increased levels of BCAAs predict both type 1 and 2 diabetes [22], [41], [42] and have been shown to contribute to insulin resistance [43]. An abnormal insulin response caused by increased levels of BCAAs could thus potentially explain the reduced glycaemic control in GF NOD mice.

We also observed an increase in the amino acids glycine, phenylalanine, tyrosine and 2-hydroxybutyric acid in GF versus CONV-R NOD mice, which was not seen in the previous study in GF versus CONV-R Swiss Webster mice [31]. These metabolites are associated with progression to insulin resistance and type 2 diabetes [41], [42], [44] but it is unclear whether they also affect beta cell function.

The differences in the metabolic profiles between GF and CONV-R NOD mice in our study correlated significantly with differences in the metabolic profiles between newborn infants who later progressed to type 1 diabetes and non-progressors from a previous study [22]. Notably, elevated insulinotropic BCAAs and diminished TCA cycle metabolites had been observed in these pre-diabetic children even before the first seroconversion for islet autoantibodies [22]. These results indicate that the changes in metabolic profiles in infants who later progress to type 1 diabetes could be microbially driven.

In our study, the effects of the microbiota in NOD mice were similar in males and females and thus not likely to be dependent on testosterone levels, in contrast to previous results that identified a gender-dependent protective effect of the microbiota in NOD mice [21]. This is of importance because type 1 diabetes does not have a female bias in humans [3] and suggests that there are microbial effects in addition to those mediated by sex hormones that may protect against autoimmune diabetes.

In conclusion, our results support a role of the gut microbiota as a regulator of diabetic autoimmunity and glucose metabolism and suggest that altered microbial metabolism may contribute to the pathogenesis of type 1 diabetes.

## Supporting Information

Figure S1 **Beta cell area in 23 week old NOD mice.** Insulin area as a percent of total pancreas area was measured in female mice at 23 weeks (n = 6 per group). Data are presented as mean ± s.e.m.
(EPS)

Figure S2 **Altered IAA titers in GF NOD mice.** Scatter plot of relative IAA units (4w GF n = 17, CONV-R n = 18; 9w GF n = 14, CONV-R n = 21; 23w GF n = 20, CONV-R n = 16). Line represents median value **p<0.01 determined by Student's *t*-test.
(EPS)

Figure S3 **Glucose tolerance in GF NOD mice.** Oral glucose tolerance test, average area under the curve (AUC) and serum insulin levels in GF and CONV-R mice at 17–18 weeks of age (n = 4–5). Data are presented as mean ±s.e.m. *p<0.05 determined by Student's *t*-test.
(EPS)
